# Improving personalized federated learning to optimize site-specific performance in computer-aided detection/diagnosis

**DOI:** 10.1117/1.JMI.12.5.054503

**Published:** 2025-10-22

**Authors:** Aiki Yamada, Shouhei Hanaoka, Tomomi Takenaga, Soichiro Miki, Takeharu Yoshikawa, Osamu Abe, Toshiya Nakaguchi, Yukihiro Nomura

**Affiliations:** a Chiba University, Graduate School of Science and Engineering, Department of Medical Engineering, Chiba, Japan; b The University of Tokyo Hospital, Department of Radiology, Tokyo, Japan; c The University of Tokyo Hospital, Department of Computational Diagnostic Radiology and Preventive Medicine, Tokyo, Japan; d Chiba University, Center for Frontier Medical Engineering, Chiba, Japan

**Keywords:** computer-aided detection/diagnosis, federated learning, personalized federated learning, cerebral aneurysm, brain metastasis, liver lesion

## Abstract

**Purpose:**

Personalized federated learning (PFL) has been explored to address data heterogeneity while preserving privacy, and its application in computer-aided detection/diagnosis (CAD) software has been investigated. Ditto, a commonly studied PFL method, trains global and personalized models but is limited by instability in model updates and high hyperparameter tuning costs. We proposed Improved Ditto, a PFL method that dynamically adjusts the proportion of global model weights during personalized model updates to enhance stability and reduce hyperparameter tuning costs.

**Approach:**

We introduced a personalized model update rule in Improved Ditto that dynamically determines the proportion of global model weights based on the L2-norm of the gradient-derived and global-model-derived terms. This method was evaluated using three types of CAD software: cerebral aneurysm detection in magnetic resonance (MR) angiography images (segmentation), brain metastasis detection in contrast-enhanced T1-weighted MR images (object detection), and liver lesion classification in gadolinium-ethoxybenzyl-diethylenetriamine pentaacetic acid-enhanced MR images (classification). The proposed method was compared with several conventional methods.

**Results:**

In two out of three CAD software, the performance of Improved Ditto was competitive with Ditto and other federated-learning-based methods. The proposed method achieved a narrower hyperparameter search space, which contributed to reducing the tuning costs. In addition, it improved the stability of personalized model updates, suggesting enhanced adaptability to diverse datasets and tasks.

**Conclusions:**

We demonstrate that dynamically adjusting global model weights during personalized model updates can improve the stability and adaptability of PFL. The proposed method reduces the hyperparameter tuning costs and offers potential benefits for CAD software.

## 1 Introduction

Several research groups have developed computer-aided detection/diagnosis (CAD) software. Machine learning, including deep learning, has been used as the primary technology in recent years.^1^^–^^5^ The performance of CAD software depends on the quality and quantity of data used to train machine learning models. If the data characteristics during development and practical use differ, the CAD software may perform sub-optimally. In addition, differences in scanners or scan parameters between sites (e.g., hospitals) and differences in subjects due to population characteristics influence data heterogeneity.^6^ Recent guidelines have recommended using data from multiple sites to develop artificial intelligence-based medical image processing.^7^^,^^8^ Various groups, including ours, have demonstrated that training using data from multiple sites enhances the generalization ability of CAD software for external datasets.^9^^–^^12^

CAD software development strategies using data from multiple sites typically use a centralized strategy (hereafter referred to as “Centralized”) (Fig. 1), where anonymized patient data are collected from multiple sites and processed at a central development institution.^13^ However, developing CAD software using Centralized is limited by privacy concerns, regulatory restrictions, and various legal, ethical, and technological barriers to data gathering.^14^ Recently, federated learning (FL)^15^ (Fig. 2) has gained considerable attention. It is a framework that trains a shared model (hereafter referred to as “global model”) from multiple decentralized sites (hereafter referred to as “clients”) without sharing their data. In FL, a single global model is concurrently trained across multiple clients, with model updates aggregated on a central site (hereafter referred to as “server”) to renew a global model that reflects the data from all participating clients.

**Fig. 1:**
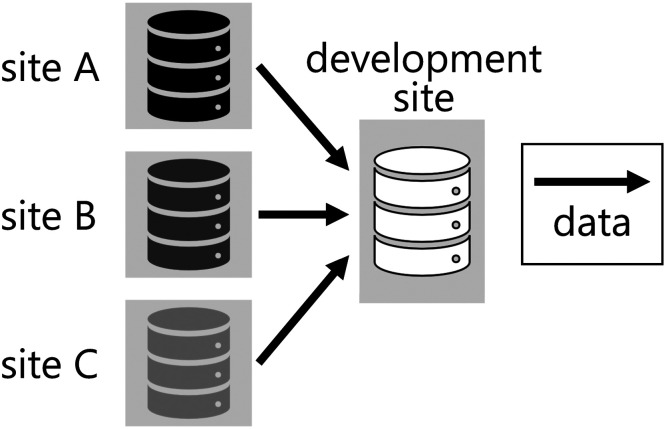
Structure of Centralized.

**Fig. 2:**
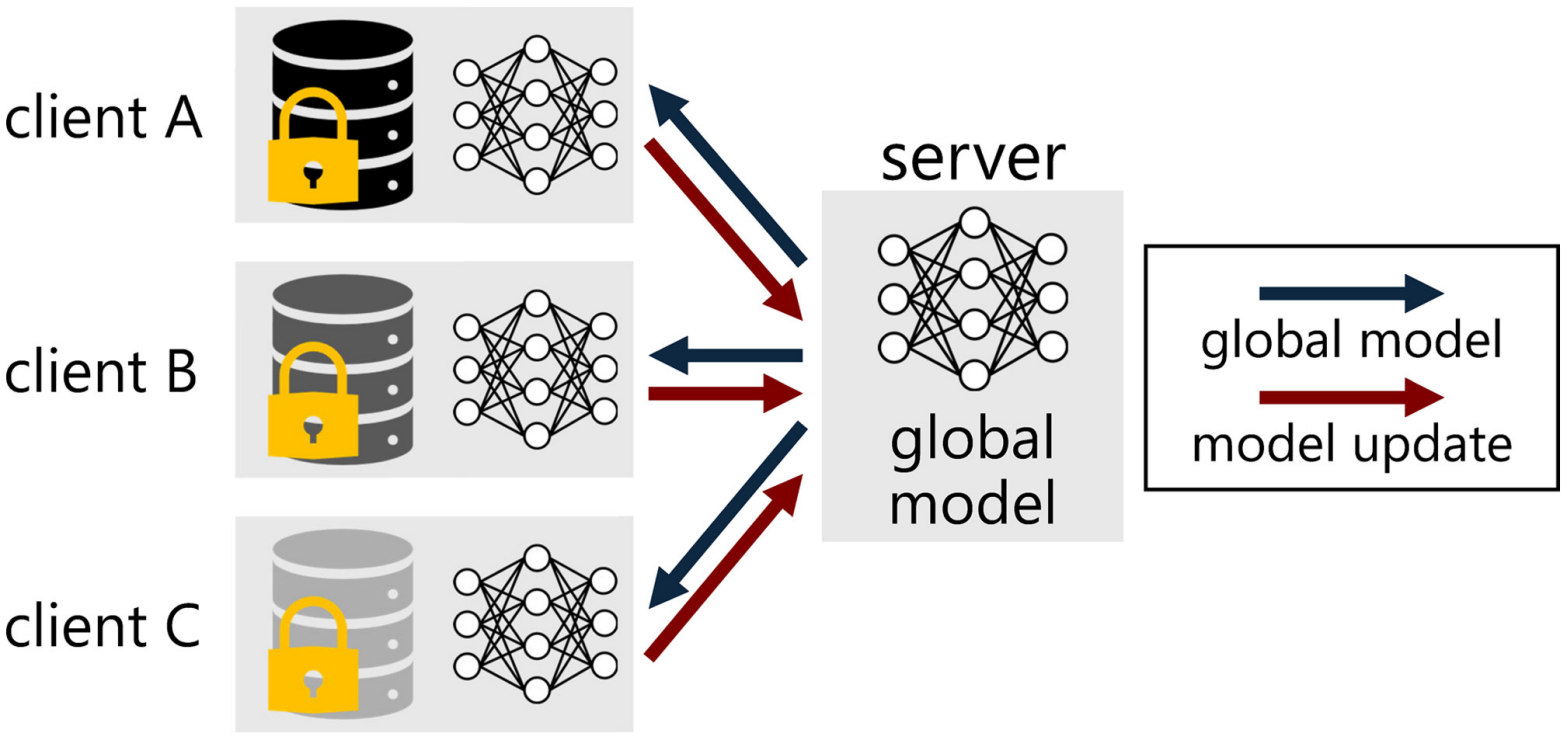
Structure of federated learning.

However, vanilla FL cannot build a client-specific model that maximizes the performance for the client. That causes difficulties when the distribution differs across clients. In this case, applying the obtained global model directly to each client may be suboptimal.^16^^,^^17^ Therefore, in addition to training a widely applicable global model, we assert that training each client-specific model to optimize the entire performance of CAD software is crucial.^16^^,^^18^

An ideal approach to develop widely applicable personalized FL (PFL), which focuses on learning client-specific models (hereafter referred to as “personalized models”), should be: (1) applicable to various CAD applications to improve client-specific performance, (2) compatible with various deep learning architectures, tasks (e.g., segmentation, object detection, and classification), data dimensions, and loss functions, and (3) minimized or eliminated hyperparameter tuning cost. Building hyperparameter-free methods is challenging; nonetheless, approaches that reduce tuning costs are highly desirable especially because established tuning strategies for noncentralized settings remain limited.

However, existing PFL methods^17^^,^^19^^–^^25^ do not meet these criteria. For instance, Per-FedAvg^20^ uses model-agnostic meta-learning^26^ to learn a global model that can be quickly adapted to the data of each client. After training the global model, each client fine-tuned it using a few gradient steps. However, this method requires tuning separate learning rates for global and personalized model updates, thereby increasing the cost and complexity of hyperparameter tuning. By contrast, Ditto^22^ simultaneously maintains a global model and client-specific personalized models, where global model weights are used to update personalized models. However, Ditto uses a sensitive hyperparameter to update the personalized models using global model weights. This hyperparameter considerably affects overall performance. The optimal value of this hyperparameter can differ widely, depending on the client or CAD application, making tuning necessary and expensive. Similar to Ditto, in adaptive personalized federated learning (APFL),^21^ each client maintains both a global and a personalized model. The personalized models are updated as a weighted combination of the global and personalized models. However, as in Ditto, APFL also faces challenges in tuning the mixing hyperparameter that controls this combination, which plays a crucial role in balancing generalization and personalization and can significantly impact the overall performance. Likewise, FedPer^19^ and FedRep^17^ attempt to enhance personalization by splitting the model into a shared body (across clients) and a client-specific head. However, these strategies limit the construction of a generalizable model. Moreover, FedBABU^25^ splits the model into a shared body and a fixed head. Although this model has demonstrated strong performance in classification tasks, its effectiveness in other tasks, such as object detection or segmentation, has not been established, thereby reducing its overall robustness and performance across diverse tasks and datasets.

In this study, we propose Improved Ditto to overcome these theoretical challenges. It can address several limitations of the original Ditto, including instability in personalized model updates, fixation of the hyperparameter lambda during personalized model updates throughout training, and the high hyperparameter tuning cost. The personalized model update rule is changed in Improved Ditto to dynamically determine the proportion of global model weights. The key contributions of this study are as follows.

• We demonstrate that the personalized model update rule in Ditto can lead to model update instability, providing a theoretical explanation for this issue.
• Improved Ditto addresses this issue by ensuring stable model updates through a dynamic weight adjustment mechanism.
• This adaptive strategy also reduces the hyperparameter tuning cost and improves process efficiency.
• We conducted experiments using three types of CAD software to demonstrate the versatility and robustness of the proposed method.

## 2 Background

### 2.1 Federated Learning

In FL, the server sends a global model to each client, and the clients train the global model using their data. Subsequently, clients send updates of the trained global model back to the server. Finally, the server combines the model updates to generate a new global model, and one process of this flow is defined as one round. We used federated averaging (FedAvg)^15^ to update the global model as follows:

$${𝔀}^{t+1}={𝔀}^{t}-\sum_{k=1}^{K}\frac{N_{k}}{N}\nabla F_{k}({𝔀}_{t}^{k}),\;N=\sum_{k=1}^{K}N_{k},$$ (1)

where t is the index of the training rounds, k represents the index of the clients, K is the total number of clients, ${𝔀}^{t}$ denotes the weight of the global model in round t, ${𝔀}_{k}^{t}$ is the weight of client k after training for several epochs in round t, $F_{k}({𝔀}_{t}^{k})$ is the local objective (loss function) for client k, $N_{k}$ is the number of training data samples at client k, and N is the total number of training samples across all clients.

### 2.2 Personalized Federated Learning

PFL trains client-specific models using data collected from multiple distributed sites, enabling adaptation to the data characteristics of each site and improving the overall performance. PFL has also been used in medical image processing.^27^^,^^28^

#### 2.2.1 Ditto

Ditto is a PFL method that simultaneously trains global and personalized models for each client (Fig. 3). The weights of the global model (${𝔀}^{*}$) are iteratively used to update each personalized model. Ditto is model agnostic and can be applied to various deep learning models, making it highly versatile for diverse applications.

**Fig. 3:**
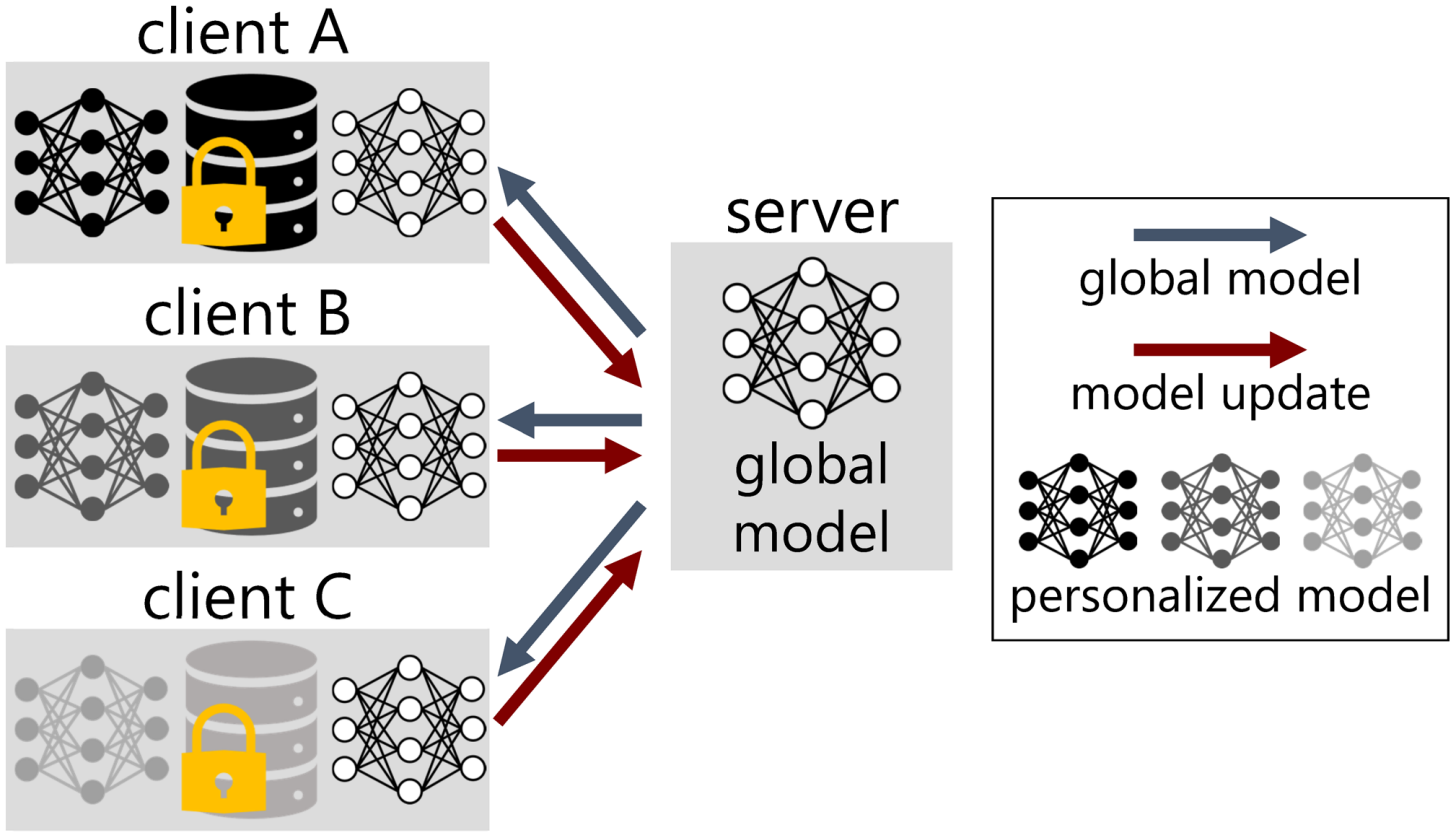
Structure of Ditto/Improved Ditto.

Algorithm 1 presents the training procedure of Ditto. The personalized model update formula used by Ditto is as follows:

$$v_{k}^{t+1}=v_{k}^{t}-\eta (\nabla F_{k}(v_{k}^{t})+\lambda (v_{k}^{t}-{𝔀}^{t})),$$ (2)

where $v_{k}^{t}$ represents the weights of the personalized model for client k in round t, $F_{k}(v_{k}^{t})$ is the local objective (loss function) for client k, η denotes the learning rate, and λ is a hyperparameter that controls the weights of the two terms $\nabla F_{k}(v_{k}^{t})$ and $\left(v_{k}^{t}-{𝔀}^{t}\right)$. The first term is an ordinal gradient descent term, and the second term forces each personalized model into similarity to the global model. We use FedAvg [Eq. (1)] for the global model update in this study. Notably, the global model training is independent of each personalized model training.

**Algorithm 1:** Training procedure of Ditto.

Algorithm 1: Ditto for personalized FL
Parameter K: the total number of clients, T: the total number of rounds, F: loss function, η: the learning rate of optimizer, ${𝔀}^{*}$: the weights of the global model, $v_{k}^{t}$: the weights of the personalized model for client k in round t, $E_{\text{global}}$: the number of epochs for training the global model, $E_{\text{personalized}}$: the number of epochs for training the personalized models, λ: the hyperparameters for controlling the balance of the global model and the personalized models.
for t=1, …, T do
Server send ${𝔀}^{t}$ to clients
for k=1,…,K in parallel do
# update the personalized model
for $e_{p}=1,\ldots ,E_{\text{personalized}}$ do
$v_{k}^{t}≔v_{k}^{t}-\eta (\nabla F(v_{k}^{t})+\lambda (v_{k}^{t}-{𝔀}^{t}))$
# update the global model using selected optimizer (e.g., SGD)
for $e_{g}=1,\;\ldots ,E_{\text{global}}$ do
${𝔀}_{k}^{t}\leftarrow GLOBAL\_MODEL\_TRAIN({𝔀}^{t},\nabla F({𝔀}^{t}))$
Send $\Delta_{k}^{t}≔{𝔀}_{k}^{t}-{𝔀}^{t}$ back to server
Server received $\Delta_{k\in [K]}^{t}$
# update the global model at server (e.g., FedAvg)
${𝔀}_{k}^{t}\leftarrow GLOBAL\_MODEL\_TRAIN({𝔀}^{t},\Delta_{k\in [K]}^{t})$
return $\{v_{k}^{T}{\}}_{\in [K]}$, ${𝔀}^{T}$

#### 2.2.2 Limitations of Ditto

Ditto has a hyperparameter λ that determines the proportion of global model weights during the personalized model update. λ considerably affects the performance of personalized models. One of the limitations of Ditto is that λ remains constant throughout training, implying that it does not depend on $\nabla F_{k}(v_{k}^{t})$ (hereafter referred to as the “gradient term”) as well as $\left(v_{k}^{t}-{𝔀}^{t}\right)$ (hereafter referred to as the “proximal term”) in Eq. (2). In addition, the optimal value varies with the client or the CAD software, increasing the adjustment cost. Furthermore, the optimal value of λ may change when the magnitude of the loss function changes. Therefore, the optimal solution changes when the loss function is multiplied by a constant value. This limitation is discussed in detail below.

First, we considered an ordinal deep learning algorithm (not FL) with gradient descent. Suppose that the loss function is scaled by a factor c (a positive constant), let the new loss function be defined as:

$$F^{\prime }(𝔀)≔c\cdot F(𝔀).$$ (3)

This modification does not change the stationary points of the loss function F; therefore, it does not affect the optimal result of the gradient descent algorithm. This is explained below. The first-order partial derivative of F becomes

$$\nabla F^{\prime }(𝔀)=\nabla (c\cdot F(𝔀))=c\cdot \nabla F(𝔀).$$ (4)

Substituting this into a standard gradient descent update rule gives

$${𝔀}^{t+1}={𝔀}^{t}-\eta \nabla F^{\prime }({𝔀}^{t})={𝔀}^{t}-(c\cdot \eta )\nabla F({𝔀}^{t}).$$ (5)

The magnitude of the gradient vector “$\nabla F({𝔀}^{t})$” is scaled by c; however, its direction remains unchanged. In standard deep learning models, parameters are updated in the direction indicated by the gradient, using gradient descent-based methods. The target minimum remains the same, provided the gradient direction remains unchanged; thus, the final result theoretically remains unaffected.

Subsequently, we consider the personalized model update in Ditto. Unlike the standard one, the updated formula includes a proximal term. Suppose the loss function is scaled by c; the personalized model update formula of Ditto becomes

$$v_{k}^{t+1}=v_{k}^{t}-\eta (c\cdot (\nabla F_{k}(v_{k}^{t}))+\lambda (v_{k}^{t}-{𝔀}^{t})).$$ (6)

Compared with Eq. (2), only the gradient term is affected by the constant c, leading to a change in the direction of the weight update vector, which is scaled by the learning rate (η) and determines the direction of the model update. Consequently, this may cause numerical optimization to converge at a different solution, altering the performance of the personalized model. In addition, this introduces sensitivity of the optimal value of λ to nonessential scaling of the loss function, making the tuning process complex.

## 3 Proposed Methods

In this section, we outline our proposed method, Improved Ditto (Fig. 3), and provide a better understanding of its advantages over Ditto.

### 3.1 Improved Ditto

We proposed a new update formula that dynamically adjusts λ based on the magnitude of the gradient term ($\nabla F_{k}(v_{k}^{t})$) and the proximal term ($v_{k}-{𝔀}^{t}$). The proposed personalized update formula is as follows:

$$v_{k}^{t+1}=v_{k}^{t}-\eta (\nabla F_{k}(v_{k}^{t})+𝓇\frac{\|\nabla F_{k}(v_{k}^{t}){\|}_{2}}{\|v_{k}^{t}-{𝔀}^{t}{\|}_{2}}(v_{k}^{t}-{𝔀}^{t})),$$ (7)

where 𝓇 is a new hyperparameter that controls the weight ratio between the personalized and global models. The weights of all the layers within both models were vectorized (i.e., flattened and concatenated) applied before calculating the L2-norm of the proximal term $\|v_{k}^{t}-{𝔀}^{t}{\|}_{2}$.

### 3.2 Effect of Scaling the Loss Function in Improved Ditto

Let us consider the case in which the loss function in the Improved Ditto update formula is scaled by constant c. The L2-norm of the scaled gradient term is calculated by vectorizing the model weights as follows:

$$\|\nabla F_{k}^{\prime }(v_{k}^{t}){\|}_{2}=\|\nabla (c\cdot F_{k}(v_{k}^{t})){\|}_{2}\;=c\|\nabla F_{k}(v_{k}^{t}){\|}_{2}.$$ (8)

Based on these results and Eq. (5), the updated formula for the personalized model when the loss function is scaled by a constant c is derived as follows:

$$v_{k}^{t+1}=v_{k}^{t}-\eta (\nabla F_{k}^{\prime }(v_{k}^{t})+⏧\frac{\|\nabla F_{k}^{\prime }(v_{k}^{t}){\|}_{2}}{\|v_{k}^{t}-{⏧}^{t}{\|}_{2}}(v_{k}^{t}-{⏧}^{t}))\;=v_{k}^{t}-\eta (c\cdot \nabla F_{k}(v_{k}^{t})+⏧\frac{c\cdot \|\nabla F_{k}(v_{k}^{t}){\|}_{2}}{\|v_{k}^{t}-{⏧}^{t}{\|}_{2}}(v_{k}^{t}-{⏧}^{t}))\;=v_{k}^{t}-c\cdot \eta (\nabla F_{k}(v_{k}^{t})+⏧\frac{\|\nabla F_{k}(v_{k}^{t}){\|}_{2}}{\|v_{k}^{t}-{⏧}^{t}{\|}_{2}}(v_{k}^{t}-{⏧}^{t})).$$ (9)

Compared with Eq. (7), the gradient and proximal terms are equally influenced by the constant c. Therefore, the direction of the weight update vector in the new update formula [Eq. (9)], $\left(\nabla F_{k}(v_{k}^{t})+𝓇\frac{\|\nabla F_{k}(v_{k}^{t}){\|}_{2}}{\|v_{k}^{t}-{𝔀}^{t}{\|}_{2}}(v_{k}^{t}-{𝔀}^{t})\right)$, remains identical to that in the original formula. Therefore, ideally or explicitly, by scaling the learning rate η by 1/c, the update path is unaltered, and the model converges to the same local minimum. Consequently, parameter 𝓇 may be easier to adjust compared with λ. In addition, considering “$\frac{\|\nabla F_{k}(v_{k}^{t}){\|}_{2}}{\|v_{k}^{t}-{𝔀}^{t}{\|}_{2}}$,” the absolute scales of the gradient and proximal terms canceled out. This ensures that the update formula processes scale invariance, thereby enabling updates independent of the scale of the loss function or models. Therefore, the newly proposed update formula, which enables dynamic adjustment using the L2-norm, effectively addresses the limitations of Ditto’s update formula.

### 3.3 Training Procedure of Improved Ditto

Algorithm 2 presents the training procedure of Improved Ditto. In this study, we used FedAvg to update the global model. We modified the update process such that the personalized model was updated without using global model weights in the first round because the initial parameters of the personalized and global models were randomly generated. In the proposed method, the updated information of the global model is transferred similarly to the FL framework. Therefore, it can be adapted to various deep learning models without depending on the model’s architecture.

**Algorithm 2:** Training procedure of Improved Ditto.

Algorithm 2: Improved Ditto for personalized FL
Parameter K, T, F, η, ${𝔀}^{*}$, $v_{k}^{t}$, $E_{\text{global}}$, $E_{\text{personalized}}$, 𝓇: the hyperparameters for controlling the balance of the global model and the personalized models.
for t=1,…,T do
Server send ${𝔀}^{t}$ to clients
for k=1,…,K in parallel do
# update the personalized model
for $e_{p}=1,\ldots ,E_{\text{personalized}}$ do
if $e_{p}=1$
$v_{k}^{t}≔v_{k}^{t}-\eta (\nabla F(v_{k}^{t}))$
else
$v_{k}^{t}≔v_{k}^{t}-\eta (\nabla F(v_{k}^{t})+𝓇\frac{\|\nabla F(v_{k}^{t}){\|}_{2}}{\|v_{k}^{t}-{𝔀}^{t}{\|}_{2}}(v_{k}^{t}-{𝔀}^{t}))$
# update the global model using selected optimizer (e.g., SGD)
for $e_{g}=1,\ldots ,E_{\text{global}}$ do
${𝔀}_{k}^{t}\leftarrow GLOBAL\_MODEL\_TRAIN({𝔀}^{t},\nabla F({𝔀}^{t}))$
Send $\Delta_{k}^{t}≔{𝔀}_{k}^{t}-{𝔀}^{t}$ back to server
Server received $\Delta_{k\in [K]}^{t}$
# update the global model at server (e.g., FedAvg)
${𝔀}_{k}^{t}\leftarrow GLOBAL\_MODEL\_TRAIN({𝔀}^{t},\Delta_{k\in [K]}^{t})$
return $\{v_{k}^{T}{\}}_{\in [K]}$, ${𝔀}^{*}$

## 4 Experiments

This study was approved by the ethics review boards of our institutions. We targeted three types of CAD applications to evaluate the generalizability of the proposed method; (1) cerebral aneurysm detection in magnetic resonance (MR) angiography (MRA) images using a three-dimensional (3D) U-Net^29^-based algorithm, (2) brain metastasis detection in contrast-enhanced T1-weighted MR images using a single shot multibox detector (SSD)-based algorithm,^30^ and (3) liver lesion classification in gadolinium-ethoxybenzyl-diethylenetriamine pentaacetic acid (Gd-EOB-DTPA)-enhanced MR images using a 3D ResNet-18^31^ with a convolutional block attention module (CBAM)^32^-based algorithm. Based on previous research, each task was assigned a distinct category: cerebral aneurysm detection as segmentation, brain metastasis detection as object detection, and liver lesion classification as classification. Appendices A, B, and C provide detailed descriptions of datasets, CAD software algorithms, and hyperparameter tuning methods (other than λ and 𝓇).

### 4.1 Training the Three Types of CAD Software

We compared the following ten methods for each CAD software:

1. Centralized (baseline)
2. training using only data from each client (hereafter referred to as EC)
3. FL
4. FL + (partial) fine-tuning (FL+FT): Fine-tuning specific parts of the model for each client using the model trained in (3) FL as the initial parameter
5. FL + full fine-tuning: Fine-tuning the entire model for each client using the model trained in 3) FL as the initial parameter
6. FedRep: A PFL method that divides the model into a head and body. The body and head are trained in an alternating manner at each client.
7. FedBABU: A PFL method that divides the model into a head and body; only the body is trained at each client.
8. APFL: A PFL method that maintains both the global model and the personalized model for each client. The personalized model is constructed as a weighted combination of the global model and local models.
9. Ditto
10. Improved Ditto (proposed method).

In the FL-based methods, we used hyperparameters (e.g., the learning rate) optimized in hyperparameter tuning in the Centralized setting, excluding the hyperparameters in Appendix D. We used the competition performance metric (CPM)^33^ as the evaluation criterion for cerebral aneurysm and brain metastasis detection. This metric provides a single-number comparison of the free-response receiver operation characteristic (FROC) curves across methods by defining the average sensitivity for a predefined false positive (FP)/case (1/8, 1/4, 1/2, 1, 2, 4, or 8 FPs/case) along the FROC curves. Accuracy was used as the evaluation criterion for liver lesion classification.

The values of λ and 𝓇, which determine the weights of the global model’s parameters in updating the personalized model, are important hyperparameters in this study. Therefore, we used a grid search to optimize their values and examined the following 20 candidates: 0.01, 0.025, 0.033, 0.05, 0.75, 0.1, 0.25, 0.33, 0.5, 0.75, 1, 1.5, 2, 5, 10, 20, 30, 50, 70, and 100. Appendix D describes the other hyperparameters for each method.

## 5 Results

### 5.1 Cerebral Aneurysm Detection

Table 1 presents the CPM scores of the training strategies for cerebral aneurysm detection, and Fig. 4 illustrates the FROC curves for cerebral aneurysms. The highest CPM scores for datasets A and B were obtained via Centralized. The highest CPM score for dataset C was obtained using FL + FT (the last block). Table 2 details the CPM for each λ value (Ditto), and Table 3 presents CPM scores for each 𝓇 value (Improved Ditto).

**Table 1:** CPM score of each method used for cerebral aneurysm detection.

Method		Dataset A	Dataset B	Dataset C	Average
Centralized		**0.840**	**1.000**	0.518	**0.786**
EC*		0.617	0.741	0.381	0.580
FL*		0.605	0.838	0.509	0.651
FL+FT*	Decoder	*0.765*	0.971	0.491	0.742
	Last block	0.655	0.838	***0.563***	0.683
	Deepest block	0.689	0.962	0.545	0.732
FL+FFT*		0.756	0.981	0.438	0.725
FedRep	Decoder	0.706	0.962	0.330	0.666
	Last block	0.555	0.571	0.446	0.524
	Deepest block	0.694	0.848	0.518	0.688
APFL (α=0.01, adaptived)		0.597	*0.990*	0.518	0.702
FedBABU	Decoder	0	0	0	0
	Last block	0	0	0	0
	Deepest block	0.706	0.961	0.509	0.726
Ditto (λ=0.1)		0.714	*0.990*	0.545	*0.750*
Improved Ditto (𝓇=0.01)		0.706	0.981	0.518	0.735

The bold text indicates the best overall method, the italic text indicates the best method except for Centralized, and the bold-italic text indicates the best method under both criteria.

EC, each client; FL, federated learning; FT, fine-tuning; FFT, full fine-tuning

**Fig. 4:**
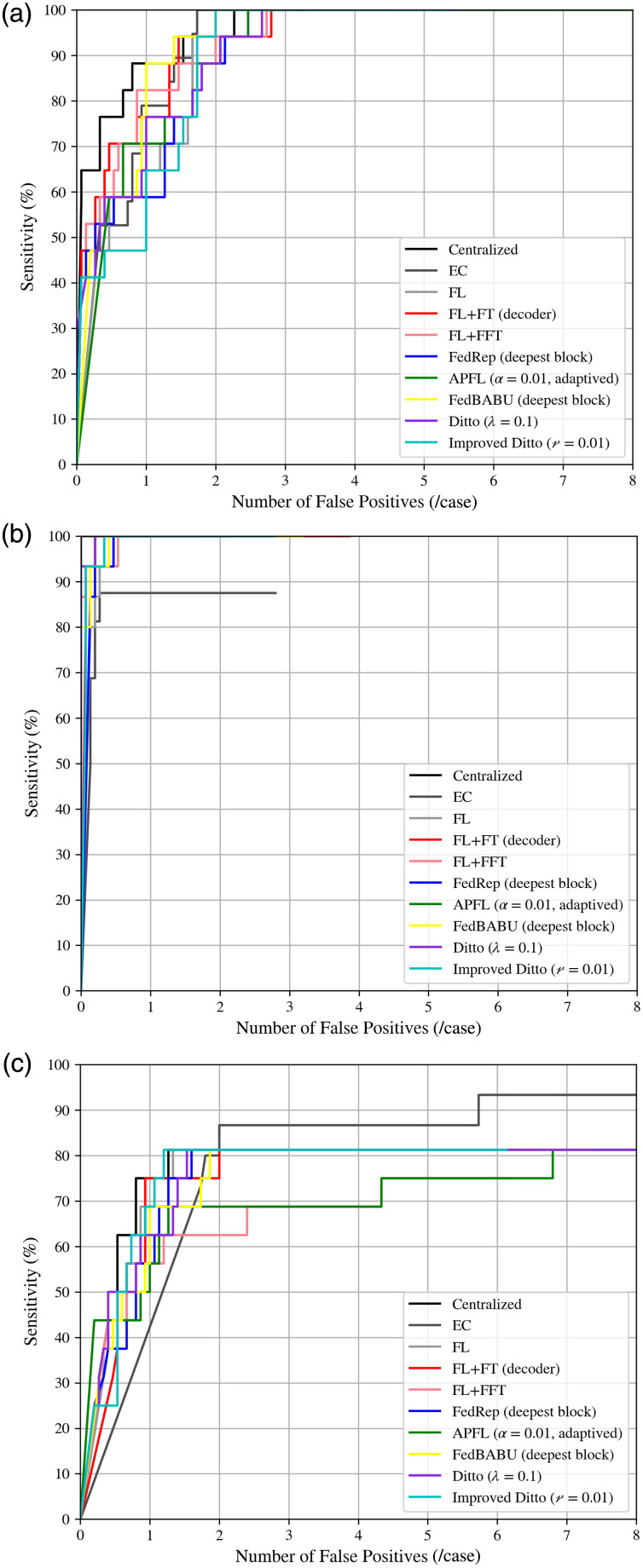
FROC curves of each method used for cerebral aneurysm detection. (a) Dataset A, (b) dataset B, and (c) dataset C.

**Table 2:** Relationship between λ of Ditto and the CPM score for each client in cerebral aneurysm detection.

λ	Dataset A	Dataset B	Dataset C	Average
0.01	0.622	0.943	0.464	0.676
0.025	0.689	0.952	0.304	0.648
0.033	0.681	0.848	0.491	0.673
0.05	0.664	**1.000**	0.429	0.697
0.075	0.622	0.990	0.509	0.707
0.1	0.714	0.990	**0.545**	**0.750**
0.25	0.740	0.952	0.482	0.725
0.33	**0.765**	0.981	0.491	0.746
0.5	0.689	0.981	0.500	0.723
0.75	0.664	0.952	0.536	0.717
1	0.613	0.981	0.491	0.695
1.5	0.647	0.981	0.509	0.712
2	0.672	0.971	0.509	0.718
5	0.689	0.981	0.491	0.720
10	0.714	0.971	0.518	0.735
20	0.622	0.848	0.518	0.662
30	0.655	0.848	0.491	0.665
50	0.639	0.848	0.527	0.671
70	0.647	0.800	0.491	0.646
100	0.655	0.848	0.509	0.671

The bold text indicates the best CPM score for each dataset and the average.

**Table 3:** Relationship between 𝓇 of Improved Ditto and CPM score for each client in cerebral aneurysm detection.

𝓇	Dataset A	Dataset B	Dataset C	Average
0.01	**0.706**	0.981	0.518	**0.735**
0.025	0.689	0.943	0.500	0.711
0.033	0.664	**0.990**	0.509	0.721
0.05	0.689	0.981	0.482	0.717
0.075	0.681	0.971	0.455	0.702
0.1	0.647	0.971	0.464	0.694
0.25	0.647	0.848	0.518	0.671
0.33	0.655	0.838	0.473	0.656
0.5	0.655	0.848	0.413	0.639
0.75	0.647	0.838	0.464	0.650
1	0.639	0.838	0.455	0.644
1.5	0.630	0.838	**0.545**	0.671
2	0.630	0.971	0.536	0.712
5	0.0	0.0	0.0	0.0
10	0.0	0.0	0.0	0.0
20	0.0	0.0	0.0	0.0
30	0.0	0.0	0.0	0.0
50	0.0	0.0	0.0	0.0
70	0.0	0.0	0.0	0.0
100	0.0	0.0	0.0	0.0

The bold text indicates the best CPM score for each dataset and the average.

### 5.2 Brain Metastasis Detection

Table 4 presents the CPM scores of each training strategy for brain metastasis detection, and Fig. 5 illustrates the FROC curves for brain metastasis. The highest CPM scores for datasets D and F were obtained via Ditto. The highest CPM score for dataset E was obtained using the Improved Ditto. Table 5 details the CPM score for each λ value (Ditto), whereas Table 6 presents that of each 𝓇 value (Improved Ditto).

**Table 4:** CPM score for each method used for brain metastasis detection.

Method	Dataset D	Dataset E	Dataset F	Average
Centralized	0.589	0.634	0.633	0.619
EC*	0.597	0.424	0.302	0.441
FL	0.641	0.634	0.649	0.641
FL+FT*	0.576	0.668	0.514	0.586
FL+FFT*	0.558	0.756	0.604	0.640
FedRep	0.541	0.668	0.551	0.587
APFL (α=0.75)	0.662	0.752	0.584	0.666
FedBABU	0.493	0.529	0.551	0.524
Ditto (λ=0.025)	***0.688***	0.693	***0.722***	0.701
Improved Ditto (𝓇=0.1)	0.667	***0.862***	0.580	***0.703***

The bold text indicates the best overall method, the italic text indicates the best method except for Centralized, and the bold-italic text indicates the best method under both criteria.

EC, each client; FL, federated learning; FT, fine-tuning; FFT, full fine-tuning.

**Fig. 5:**
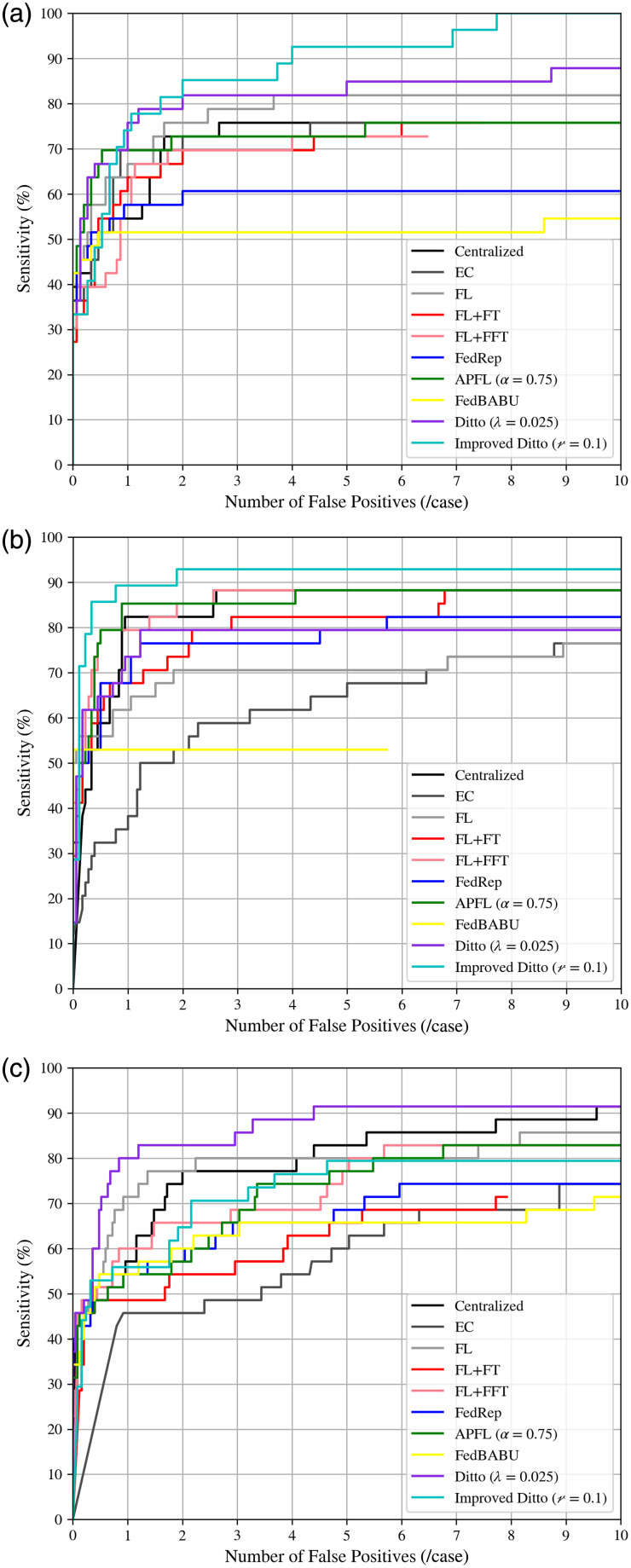
FROC curves of each method used for brain metastasis detection. (a) Dataset D, (b) dataset E, and (c) dataset F.

**Table 5:** Relationship between λ of Ditto and the CPM score for each client in brain metastasis detection.

λ	Dataset D	Dataset E	Dataset F	Average
0.01	0.571	0.718	0.661	0.650
0.025	**0.688**	0.693	0.722	**0.701**
0.033	0.619	0.664	0.645	0.643
0.05	0.645	0.672	0.620	0.646
0.075	0.623	0.723	0.718	0.688
0.1	0.654	0.710	0.596	0.653
0.25	0.610	0.735	0.612	0.653
0.33	0.602	0.618	**0.784**	0.668
0.5	0.589	0.651	0.629	0.623
0.75	0.584	0.613	0.629	0.609
1	0.602	0.681	0.641	0.641
1.5	0.589	0.639	0.673	0.634
2	0.628	0.681	0.649	0.653
5	0.623	0.710	0.694	0.676
10	0.641	0.727	0.616	0.661
20	0.580	0.714	0.555	0.617
30	0.584	0.752	0.684	0.677
50	0.576	**0.756**	0.629	0.654
70	0.576	0.752	0.620	0.649
100	0.589	**0.756**	0.633	0.652

The bold text indicates the best CPM score for each dataset and the average.

**Table 6:** Relationship between 𝓇 of Improved Ditto and the CPM score for each client in brain metastasis detection.

𝓇	Dataset D	Dataset E	Dataset F	Average
0.01	0.658	0.706	0.653	0.672
0.025	0.584	0.685	0.669	0.646
0.033	0.623	0.693	0.633	0.650
0.05	0.563	0.689	0.657	0.636
0.075	0.571	0.613	**0.673**	0.619
0.1	**0.667**	**0.862**	0.580	**0.703**
0.25	0.606	0.689	0.600	0.632
0.33	0.606	0.643	0.527	0.592
0.5	0.563	0.689	0.619	0.624
0.75	0.563	0.739	0.600	0.634
1	0.563	0.0	0.531	0.364
1.5	0.0	0.0	0.0	0.0
2	0.0	0.0	0.0	0.0
5	0.0	0.0	0.0	0.0
10	0.0	0.0	0.0	0.0
20	0.0	0.0	0.0	0.0
30	0.0	0.0	0.0	0.0
50	0.0	0.0	0.0	0.0
70	0.0	0.0	0.0	0.0
100	0.0	0.0	0.0	0.0

The bold text indicates the best CPM score for each dataset.

### 5.3 Liver Lesion Classification

Table 7 presents the accuracy of each training strategy for classifying liver lesions. Among all the sites (dataset), Ditto achieved the highest accuracy for dataset G. Conversely, FL + FT achieved the highest accuracy for dataset H. For dataset I, the highest accuracy was obtained using Improved Ditto. Table 8 illustrates the accuracy for each λ value (Ditto), whereas Table 9 presents CPM scores for each 𝓇 value (Improved Ditto).

**Table 7:** Accuracy of each method in liver lesion classification (unit, %).

Method	Dataset G	Dataset H	Dataset I	Average
Centralized	63.9	56.8	58.3	59.7
EC	58.3	49.4	55.6	54.4
FL	72.2	64.2	55.6	64.0
FL+FT	66.7	***65.4***	55.6	62.6
FL+FFT	63.9	50.6	52.8	55.8
FedRep	75.0	64.2	58.3	65.8
APFL (α=0.01, adaptived)	75.0	63.0	58.3	65.4
FedBABU	66.7	59.3	66.7	64.2
Ditto (λ=5)	***77.8***	56.8	77.2	70.6
Improved Ditto (𝓇=0.075)	75.0	61.7	***77.8***	***71.5***

The bold text indicates the best overall method, the italic text indicates the best method except for Centralized, and the bold-italic text indicates the best method under both criteria. EC, each client; FL, federated learning; FT, fine-tuning; FFT, full fine-tuning

**Table 8:** Relationship between λ of Ditto and accuracy for each client in liver lesion classification (unit, %).

λ	Dataset G	Dataset H	Dataset I	Average
0.01	52.8	55.6	41.7	50.0
0.025	55.6	53.1	55.6	54.8
0.033	55.6	55.6	55.6	55.6
0.05	61.1	53.1	47.2	53.8
0.075	55.6	59.3	52.8	55.9
0.1	58.3	56.8	55.6	56.9
0.25	63.9	58.0	61.1	61.0
0.33	61.1	55.6	58.3	58.3
0.5	61.1	58.0	63.9	61.0
0.75	72.2	56.8	66.7	65.2
1	72.2	56.8	72.2	67.1
1.5	61.1	61.7	69.4	64.1
2	77.8	53.1	75.0	68.6
5	77.8	56.8	**77.2**	**70.6**
10	72.2	58.0	72.2	67.5
20	**80.6**	60.5	69.4	70.2
30	**80.6**	58.0	72.2	70.3
50	77.8	58.0	66.7	67.5
70	75.0	**64.2**	66.7	68.6
100	72.2	56.8	61.1	63.4

The bold text indicates the highest accuracy for each dataset and the average.

**Table 9:** Relationship between 𝓇 of Improved Ditto and accuracy for each client in liver lesion classification. (unit, %).

𝓇	Dataset G	Dataset H	Dataset I	Average
0.01	61.1	55.6	61.1	59.3
0.025	61.1	59.3	61.1	60.5
0.033	66.7	61.7	69.4	65.9
0.05	72.2	60.5	72.2	68.3
0.075	75.0	61.7	**77.8**	**71.5**
0.1	72.2	**64.2**	**77.8**	71.4
0.25	75.0	56.8	66.7	66.2
0.33	**77.8**	56.8	66.7	67.1
0.5	72.2	60.5	55.6	62.8
0.75	75.0	56.8	55.6	62.5
1	72.2	50.6	61.1	61.3
1.5	72.2	61.7	52.8	62.2
2	72.2	53.1	58.3	61.2
5	55.6	49.4	38.9	48.0
10	16.7	48.1	44.4	36.4
20	16.7	48.1	47.2	37.3
30	33.3	34.6	36.1	34.7
50	16.7	18.5	19.4	18.2
70	16.7	18.5	19.4	18.2
100	16.7	18.5	19.4	18.2

The bold text indicates the highest accuracy and the average.

### 5.4 Range of Hyperparameters

Table 10 presents the values of λ or 𝓇 and the best evaluation metrics for each CAD software client. The range of λ in Ditto was [0.05, 0.33] for cerebral aneurysm detection, [0.025, 100] for brain metastasis detection, and [5, 70] for liver lesion classification, with an overall range of [0.025, 100]. By contrast, the 𝓇 ranges in Improved Ditto were [0.01, 1.5] for cerebral aneurysm detection, [0.075, 0.1] for brain metastasis detection, and [0.1, 0.33] for liver lesion classification, with an overall range of [0.01, 1.5]. These results indicate that the maximum-to-minimum ratio is 4000 for λ and 150 for 𝓇.

**Table 10:** Optimal values of λ or 𝓇 and corresponding best evaluation metrics for each CAD software client.

	CAD software	Cerebral aneurysm detection			Brain metastasis detection			Liver lesion classification		
	Dataset	A	B	C	D	E	F	G	H	I
Ditto	λ	0.33	0.05	0.1	0.025	50, 100	0.33	20, 30	70	5
	Evaluation metrics^a^	0.765	1.000	0.545	0.688	0.756	0.784	80.6	64.2	77.2
Improved Ditto	𝓇	0.01	0.033	1.5	0.1	0.1	0.075	0.33	0.1	0.075, 0.1
	Evaluation metrics^a^	0.706	0.990	0.545	0.667	0.862	0.673	77.8	64.2	77.8

a Evaluation metrics: CPM score for cerebral aneurysm and brain metastasis detection, and accuracy [%] for liver lesion classification. The largest value was selected when multiple candidate hyperparameter values yielded the best evaluation metrics.

## 6 Discussion

We proposed a novel PFL method, Improved Ditto, that addresses key limitations in existing PFL approaches and fulfills the criteria for an ideal PFL outlined in Sec. 1. Specifically, our method improves client-specific performance, reduces hyperparameter tuning costs through adaptive adjustment, and exhibits versatility across different deep learning models, tasks, and data types. Improved Ditto is an enhanced version of Ditto because it dynamically adjusts the influence of the global model during personalized updates, thereby stabilizing the training and reducing dependence on fixed hyperparameters. We demonstrated that our method improved the performance of each client through experiments using three types of CAD software. Moreover, compared with the Centralized and EC strategies, Improved Ditto achieved higher CPM scores and accuracy without requiring data sharing. These results highlight the suitability of Improved Ditto for training both personalized and generalized CAD models while preserving privacy, particularly when each site has access to sufficient computational resources.

In Improved Ditto, we extended the Ditto, which determined the ratio of the weights of the global model based on the fixed hyperparameter λ, by proposing a dynamic adjustment method that considers the ratio of the L2-norm of the gradient term ($\|\nabla F_{k}(v_{k}^{t}){\|}_{2}$) and proximal term ($\|v_{k}^{t}-{𝔀}^{t}{\|}_{2}$). The L2-norms of the models’ weights were determined by vectorizing the weights of the model. $\|\nabla F_{k}(v_{k}^{t}){\|}_{2}$ and $\|v_{k}^{t}-{𝔀}^{t}{\|}_{2}$ take on different absolute values depending on deep learning models and loss function; however, we theoretically demonstrated that using their ratio eliminates scale dependence. Consequently, Improved Ditto is versatile and applicable across various deep learning models, tasks, and dimensions of the training data.

We validated the effectiveness of the proposed method using three types of CAD software. The experimental results confirm that the proposed method improved the performance of CAD software for each client regardless of the type of task, data dimensionality (two- or three-dimensional), or the number of channels. In particular, our method outperformed existing approaches in brain metastasis detection (object detection task) and liver lesion classification (classification task). However, the performance was inferior in cerebral aneurysm detection (segmentation task) compared with the Centralized. A possible explanation is that the cerebral aneurysm dataset exhibited relatively small domain shifts across clients, which limited the benefit of personalization. Our method is particularly effective when the discrepancies in data distributions among clients are substantial, making its advantage in detecting and classifying tasks where such heterogeneity was present more apparent. Supporting this interpretation, even other PFL methods, such as Ditto and our method, underperformed in cerebral aneurysm detection. Despite this limitation, the proposed method remains adaptable to diverse client data distributions and task types; however, further improvements are required.

We compared the optimal range of the hyperparameter 𝓇 in Improved Ditto with λ in Ditto for each client. Consequently, the maximum-to-minimum ratio was 4000 for λ and 150 for 𝓇. This finding experimentally demonstrates that the range 𝓇 in Improved Ditto is significantly narrower than that of λ in Ditto. Suggesting the following advantages; First, it simplifies the initialization of the hyperparameters and reduces hyperparameter tuning cost. Second, it facilitates adaptation to new clients. In FL, a new client may join the training process, and Improved Ditto allows for a more effortless adjustment of 𝓇 compared with conventional methods. Future studies will investigate the impact of increasing the number of clients during training.

As data heterogeneity increases among clients, the performances of the models degrade. Improved Ditto mitigates this issue by dynamically adjusting the ratio between the global model and personalized models based on 𝓇. However, 𝓇 was fixed during training in this study, possibly limiting the ability of the personalized models to fully adapt to the data of each client. Consequently, to improve performance, future work will explore dynamic adjustment of 𝓇 based on data heterogeneity among clients, which is quantified using cosine similarity or Kullback–Leibler divergence. However, the computational overhead and privacy concerns associated with such similarity calculations require further investigation.

In FL-based methods, including Improved Ditto, privacy is preserved by only transferring model updates not sharing client data. However, this structure hinders efficient hyperparameter tuning, which is essential for improving model performance.^34^ Hyperparameter tuning requires testing various conditions and evaluating the performance of each model. During this process, there is a risk that the statistical characteristics of client data could be inferred from the shared update information and performance metrics.^35^ In addition, conventional methods such as grid search, random search,^36^ and Bayesian optimization^37^ involve iterative trials, leading to significantly higher computational costs and communication overhead, making them impractical. Furthermore, in environments where client data distributions differ substantially, adequate hyperparameter tuning might be impossible. In FL with differential privacy (DP),^38^^,^^39^ a key challenge lies in how model updates and performance evaluations consume the privacy budget, which represents the permissible privacy loss for safeguarding personal information. Excessive consumption, especially from tasks such as hyperparameter tuning, undermines the very privacy that DP is intended to protect. Fixing hyperparameters can mitigate information leakage; however, this is often impractical in practice. Our method narrows the tuning range and reduces adjustment costs compared to Ditto, improving efficiency under DP. Our results suggest that 𝓇=0.1 can be used as a priori standard value for most tasks. This makes it a practical solution for privacy-preserving learning, particularly in the resource-constrained real world.

Our study had several limitations. First, many hyperparameters were obtained using the FL-based method, including the proposed method, by hyperparameter tuning in Centralized. In particular, in cerebral aneurysm detection, the structure of the model is included in the hyperparameters, and the optimal architecture may vary with the method. Methods for model architecture search in FL have been proposed;^40^ however, further studies are required for their application to the proposed method and PFL. Second, the volume of image data used for each CAD software was limited. In machine learning, the effect of additional training data diminishes as the dataset increases.^41^ Pati et al.^42^ trained a deep learning model for detecting glioblastoma sub-compartment boundaries using FL with 6314 multiparametric MR images collected from 71 sites across six continents. Their findings suggested that in a federated setting, model performance does not necessarily scale proportionally or linearly with the volume of training data. Therefore, further investigation is required to better understand the relationship between the dataset size and the performance of models trained using the FL-based method in distributed learning environments. Third, our comparison focused on PFL methods that rely on local optimization strategies. We did not include methods based on fundamentally different paradigms, such as PFL using hypernetworks (pFedHN),^43^ which leverages a server-side hypernetwork to generate personalized model parameters from client-specific embeddings. Although pFedHN has shown promising results,^43^ it represents a different line of approach compared with the optimizer-based methods examined in this study. Investigating the applicability of such hypernetwork-based personalization methods remains an important direction for future work.

## 7 Conclusion

We proposed an improved PFL method that maximizes the performance of each client under several FL settings. We theoretically and experimentally demonstrated that the proposed method is applicable regardless of the dimensions of the training data, the number of channels of the deep learning models, the task of the deep learning models, and the loss function. We revealed that the parameter 𝓇, which determines the weight of the global model during personalized model updates, has a narrower range for achieving optimal site-specific performance compared with λ of Ditto. This result contributes to reducing the hyperparameter tuning cost. Our future work will include validation using other types of CAD software, the introduction of privacy-preserving techniques such as DP,^38^^,^^39^ blockchain,^44^ and homomorphic encryption^45^ to enhance data security, the proposal of an automatic adjustment method for 𝓇, and an investigation of the applicability of hypernetwork-based personalization methods.

## 8 Appendix A: Dataset of Each CAD Software

In this section, we present the datasets of each CAD software.

### 8.1 Cerebral Aneurysm Dataset

We used the same dataset as that in our previous study,^46^ which consisted of 315 MRA images from three in-hospital datasets. The cases were divided into three groups based on the scanner vendor (GE, Siemens, and Canon). The dataset details, including the annotation methods and dataset splits, are described in our previous study.^46^ Table 11 presents the population of each dataset used for cerebral aneurysm detection.

**Table 11:** Population of the cerebral aneurysm dataset.^46^

Dataset ID	A			B			C		
	Training	Validation	Test	Training	Validation	Test	Training	Validation	Test
Demographics									
No. of patients	75	15	15	75	15	15	75	15	15
No. of MRA images	75	15	15	75	15	15	75	15	15
No. of cerebral aneurysms	91	19	17	84	16	15	86	15	16

### 8.2 Brain Metastasis Dataset

We used the brain metastasis dataset from the Open Database of Tumors for Studies in Artificial Intelligence (OpenBTAI),^47^ selecting 347 contrast-enhanced T1-weighted MR studies from 75 patients, as described in our previous study.^46^ The studies were divided into three groups based on the scanner vendor (GE, Philips, and Siemens). The dataset details, including the annotation methods and exclusion criteria, are provided in our previous study.^46^ Table 12 lists the populations of each dataset used for brain metastasis detection.

**Table 12:** Population of the brain metastasis dataset.^46^

Dataset ID	D			E			F		
	Training	Validation	Test	Training	Validation	Test	Training	Validation	Test
Demographics									
No. of patients	15	5	5	13	4	5	18	4	6
No. of MR images	65	15	15	76	15	18	98	27	25
No. of brain metastases	135	27	33	91	26	34	149	27	35

### 8.3 Liver Lesion Dataset

We collected 185 Gd-EOB-DTPA-enhanced MR examinations comprising five phases (unenhanced, arterial, portal venous, equilibrium, and hepatobiliary). The MR examinations included at least one hepatocellular carcinoma (HCC), liver metastasis, or hemangioma. The target liver lesions were classified into four: these three lesions mentioned above and cysts. Among the 185 cases, 125, 31, and 29 were used for training, validation, and testing, respectively. We divided the cases into three groups based on the scanner vendor and magnetic field strength (GE 1.5 T, GE 3 T, and Siemens 3 T). Table 13 lists the details of the datasets. Table 14 presents the population of the liver lesion dataset.

**Table 13:** Details of the liver lesion dataset.

Dataset ID	MR scanners	Magnetic field strength [Tesla]	Scan parameters
G	Signa HDxt (GE Healthcare, Waukesha, United States)	1.5	Field of view (FOV), 320 to 400 mm; matrix size, 512 × 512 pixels; pixel spacing, 0.625 to 0.936 mm; slice thickness, 5 mm; repetition time (TR), 3.48 to 4.07 ms; echo time (TE), 1.64 to 1.9 ms; flip angle (FA), 15 deg
	Signa EXCITE (GE Healthcare, Waukesha, United States)	1.5	FOV, 350 to 400 mm; matrix size, 512 × 512 pixels; pixel spacing, 0.684 to 0.781 mm; slice thickness, 5 mm; TR, 3.40 to 3.52 ms; TE, 1.67 to 1.75 ms; FA, 15 deg
H	Signa HDxt (GE Healthcare, Waukesha, United States)	3.0	FOV, 320 to 400 mm; matrix size, 512 × 512 pixels; pixel spacing, 0.625 to 0.781 mm; slice thickness, 4 or 5 mm; TR, 3.15 to 3.82 ms; TE, 1.49 to 1.72 ms; FA, 12 deg
I	Skyra (Siemens Healthcare, Erlangen, Germany)	3.0	FOV, 263 to 400 mm; matrix size, 240 to 270 × 320 pixels; pixel spacing, 1.09 to 1.41 mm; slice thickness, 3 mm; TR, 3.13 to 3.87 ms; TE, 1.09 to 1.18 ms; FA, 10 deg

**Table 14:** Population of the liver lesion dataset.

Dataset	Training				Validation				Test			
	HCC	Meta	Hema	Cyst	HCC	Meta	Hema	Cyst	HCC	Meta	Hema	Cyst
G	29	65	19	48	6	13	5	11	6	13	5	12
H	67	84	39	151	15	21	11	40	15	21	8	37
I	32	34	51	60	8	4	10	18	7	3	13	13

Meta, liver metastasis; hema, hemangioma.

## 9 Appendix B: CAD Software Algorithms

In this section, we present each CAD software algorithm.

### 9.1 Cerebral Aneurysm Detection in MRA Images

In the preprocessing, the input MRA images were first resampled to a 0.3 mm isotropic voxel size using tricubic interpolation, and their signal intensity distributions were standardized using the global piecewise linear mapping.^48^ Subsequently, we extracted arterial voxels from the MRA images using the region-growing-based method, as described in our previous study.^46^ We used 3D U-Net^29^ (Fig. 6) to detect the cerebral aneurysm region.

**Fig. 6:**
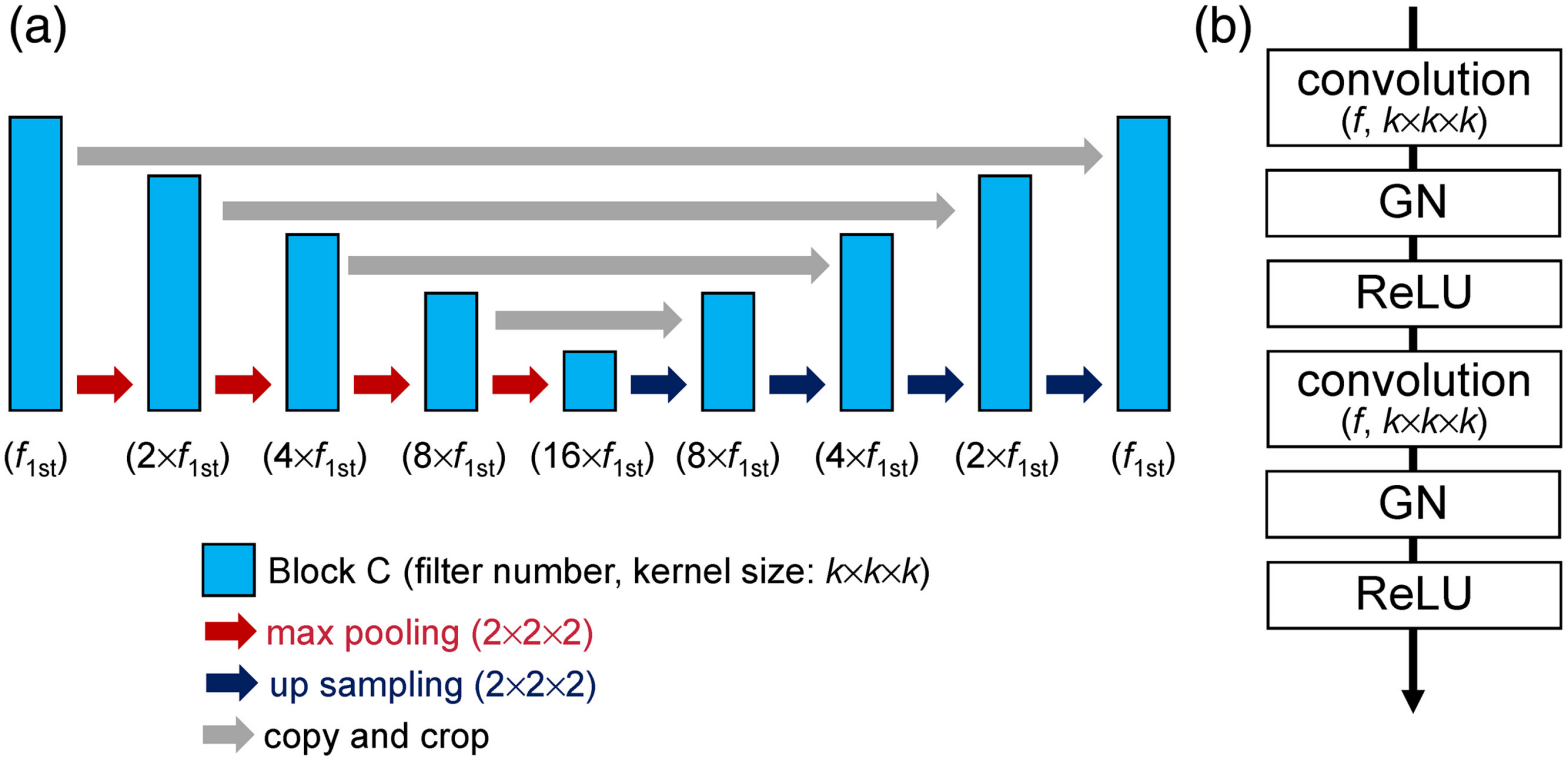
Structure of the 3D U-Net used in cerebral aneurysm detection. (a) Overview of 3D U-Net and (b) convolutional block (block C). $f_{1\mathrm{st}}$, the number of filters in the first layer; GN, group normalization with eight groups; ReLU, rectified linear unit.

In the training phase, we modified the loss function from the previously used Dice loss to DiNec loss, which is a combination of negative case max output suppression (Necmos) loss^49^ and Dice loss, enabling the inclusion of both positive cases (with the area of cerebral aneurysm) and negative cases (without the area of cerebral aneurysm, only cerebral arteries). We used stochastic gradient descent (SGD) and group normalization^50^ with eight groups in the convolution block. Regarding the training data, we extracted a 48 × 48 × 48 cubic volume of interest (VOI) from positive cases, which contain ground truth labels for cerebral aneurysms, and negative cases, which did not. In the positive cases, positive VOIs were extracted around the center of gravity of the aneurysm region for each aneurysm. Conversely, in the negative cases, negative VOIs were generated from randomly selected voxels within the preprocessed cerebral artery regions, ensuring that no aneurysm regions were included. The number of VOIs from positive and negative cases was set at a 1:1 ratio. In addition, 32 augmented VOIs were generated by random shifts within the x-, y-, and z-axes and random rotations (0, 90, 180, and 270 deg) in the axial, coronal, and sagittal planes. The augmented VOIs were changed for each epoch.

In the detection phase, cubic VOIs with a size of 48×48×48 were extracted at the lattice points within the bounding box of the segmented arterial volume, with intervals of 24 voxels in each direction. All the extracted VOIs were fed into the trained 3D U-Net model, and the outputs were combined by averaging overlapping voxels. After that, lesion candidates were identified by binarizing the combined outputs and connected component analysis.

### 9.2 Brain Metastasis Detection in Contrast-Enhanced MR Images

We used the same preprocessing, model architecture, training strategy, and detection methodology as in our previous study.^46^ Brain contrast-enhanced T1-weighted MR images were preprocessed following our established pipeline,^46^ including skull stripping with the Brain Extraction Tool,^51^ Gaussian intensity normalization,^52^^,^^53^ resampling to 1.0 mm isotropic voxels, and axial size adjustment to 256 × 256. The lesion detection model was based on the SSD architecture proposed by Zhou et al.^54^ (Fig. 7) and implemented via pytorch-ssd.^55^ The training utilized ground-truth labels, excluding small boxes. In the training phase, the ground-truth bounding boxes were derived from voxel-wise labels, excluding those with a height or width of 2 ≤ voxels. Table 15 lists the number of axial slices used to train each dataset. Each anchor (default box) was assigned a binary label based on its intersection over union (IoU) with ground-truth boxes: anchors with IoU ≥ 0.2 were labeled as positive (brain metastasis). In contrast, those with IoU < 0.2 were labeled as negative (background). We used a loss function combining the localization loss (smooth L1) and classification loss (cross-entropy). We used SGD to optimize the network weights without momentum or weight decay. In addition, two augmented slices were generated by random expansion, horizontal flip with 50% probability, and rotation (0, 90, 180, and 270 deg). The other parameters for training the model are set in previous studies.^46^^,^^54^^,^^56^ In the detection phase, each axial slice from the 3D images was fed into the trained SSD model, and the output bounding boxes from adjacent outputs were stacked to construct a 3D lesion candidate.

**Fig. 7:**
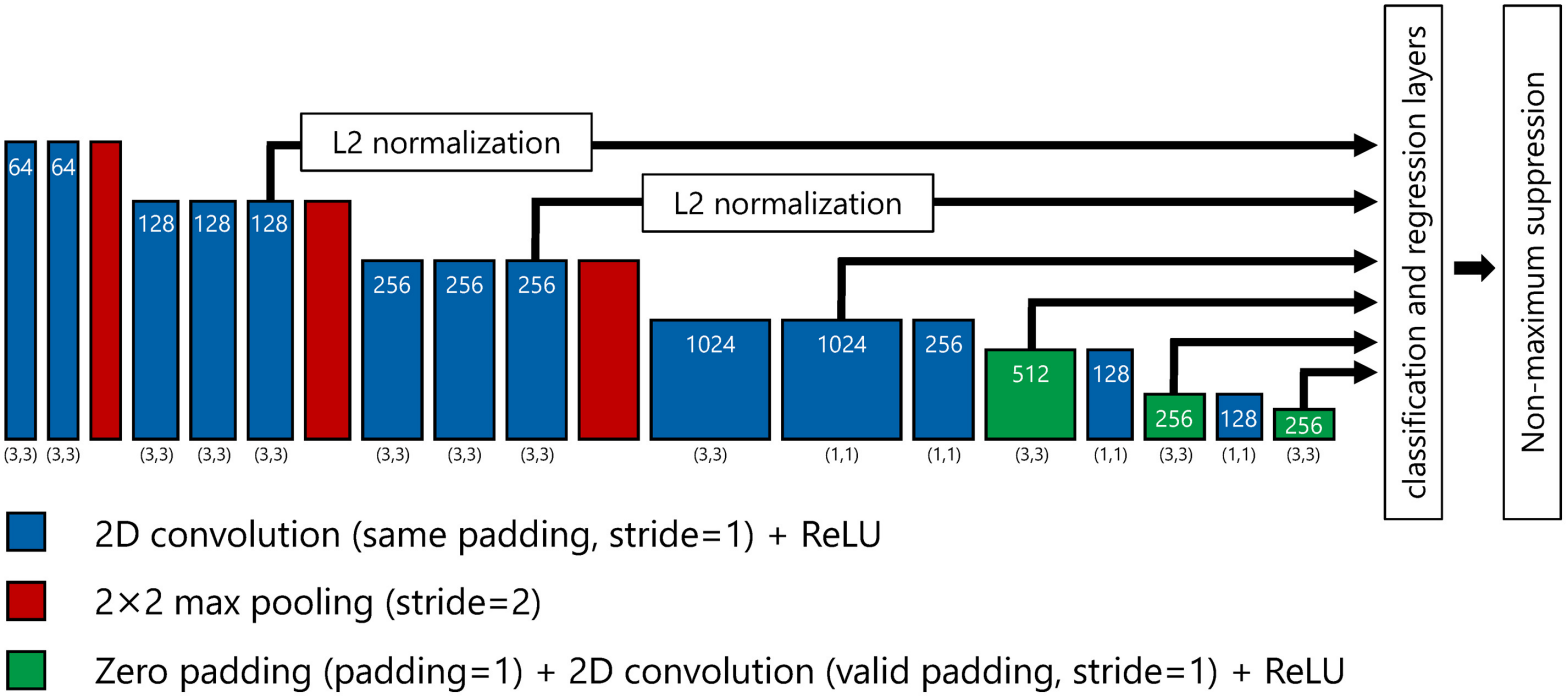
Structure of the single-shot multibox detector used in brain metastasis detection. ReLU, rectified linear unit.

**Table 15:** Number of slices used for training SSD for brain metastasis detection.^46^

Dataset ID	Training Set	Validation Set
D	1605	331
E	1514	325
F	2512	468
Total	5631	1124

### 9.3 Liver Lesion Classification in Gd-EOB-DTPA-Enhanced MR Images

In the preprocessing, groupwise registration^57^ was applied to the images in all phases for motion correction. The registration settings were determined based on a parameter file from the motion correction approach proposed by Jansen et al.^57^ Subsequently, the images were resampled to a 1.0 mm isotropic voxel size using tricubic interpolation. We used 3D ResNet-18^31^ enhanced with a CBAM^32^ as the deep learning model (hereafter referred to as 3D ResNet-18+CBAM) (Fig. 8). A preliminary version of this study was presented at the Computer Assisted Radiology and Surgery 2024 conference.^58^ In this study, we extended the previous model (3D ResNet-18) by incorporating CBAM to enhance the feature representation, which constitutes a major difference compared with the previous version. SGD was used to optimize the network weights. For each liver lesion, a 32 × 32 × 32 cubic VOI around the center of gravity of the liver lesion was extracted for each phase. The matrix size of each VOI was 5 × 32 × 32 × 32 voxels. In addition, four augmented VOIs were generated by random shifts within ±4 voxels on the x-, y-, and z-axes, random rotation (0/90/180/270 deg) in each of the axial, coronal, and sagittal planes, and random gamma correction with a magnitude (gamma value) of [0.5 to 1.5]. The augmented VOIs were changed for each epoch. In the classification phase, the extracted VOIs of size 5 × 32 × 32 × 32 were fed into the trained 3D ResNet-18+CBAM^59^^,^^60^ to obtain the classification results. In this process, each 32 × 32 × 32 VOI was input as a channel into the 3D ResNet-18+CBAM model.

**Fig. 8:**
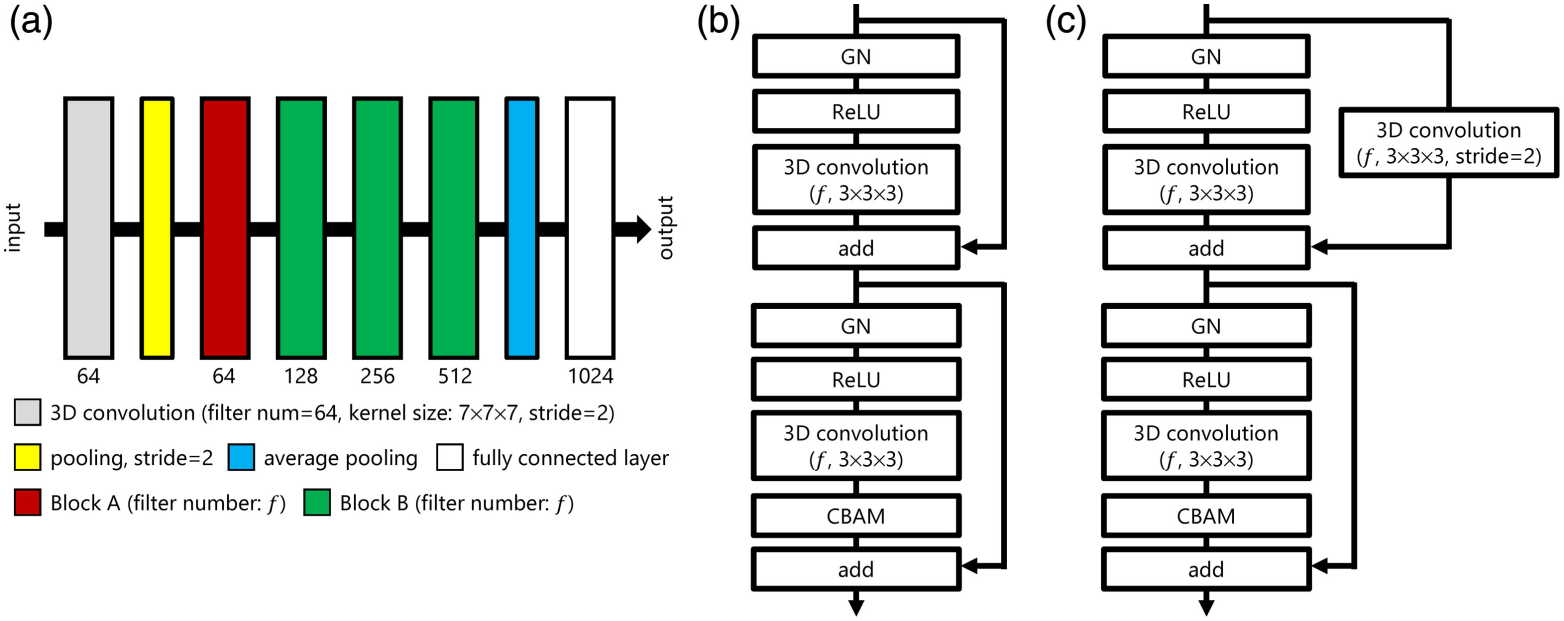
Structure of 3D ResNet-18+CBAM. (a) Overview of 3D ResNet-18+CBAM and (b), (c) residual blocks (blocks A and B). CBAM, convolutional block attention module; f, the number of filters in Block A or B; GN; group normalization with eight groups; ReLU, rectified linear unit.

## 10 Appendix C: Hyperparameter Tuning of Each CAD Software

We used a conventional strategy, where the model was trained using data collected from each site (Centralized), as a baseline. The hyperparameters for training each CAD software were optimized by hyperparameter tuning in Centralized. The model of each CAD software was implemented using Python 3.8.10 and PyTorch 1.13.0.^61^ All processes were performed on three workstations: (1) an Intel Xeon Gold 6230 2.1 GHz 20-core processor with 384 GByte RAM, NVIDIA GeForce RTX8000 GPU with 48 GByte of memory, Ubuntu 20.04.5, NVIDIA Driver 539.41.03, CUDA 11.8.0, and cuDNN 8.7.0. (2) an AMD EPYC 7742 2.25 GHz 64-core processor with 2.0 TByte RAM, NVIDIA GeForce RTX A 100 GPU with 40 GByte of memory, Ubuntu 20.04.5, NVIDIA Driver 550.90.07, CUDA 12.4, and cuDNN 8.7.0. (3) an Intel Xeon Gold 62343.3 GHz, 8-core processor with 384 GByte RAM, NVIDIA GeForce RTX A 6000 GPU with 48 GByte of memory, Ubuntu 20.04.5, NVIDIA Driver 535.161.08, CUDA 12.2, and cuDNN 8.7.0. A single GPU was used to train the models.

### 10.1 Cerebral Aneurysm Detection

We used 50 trials of hyperparameter tuning with a random search.^36^ The area under the curve value of the FROC curve, with an upper limit of two false positives (FPs) per case, was an evaluation criterion. The tuned hyperparameters were the learning rate of the SGD ($10^{-4}$ to $10^{-2}$), the batch size (2 to 64, step: 2), the number of μ of the DiNec loss ($10^{-4}$ to 1), the depth of 3D U-Net (4/5), the number of filers in the first convolution layer ($f_{1\mathrm{st}}$, 8 to 64, step: 8), and the batch size (2 to 64, step: 2). The number of epochs was set to 100. In the performance evaluation, a lesion candidate was classified as a true positive (TP) if it satisfied at least one of the outlined criteria, which is consistent with the criteria used in previous studies.^11^^,^^46^ These judgments were automatically processed.

1. The distance between the center of gravity of the lesion candidate and the center of gravity or location of the lesion center of any aneurysm was less than the radius of the aneurysm.
2. The distance between the center of gravity of the lesion candidate and the center of gravity or location of the lesion center of any aneurysm was <3.0  mm.

### 10.2 Brain Metastasis Detection

We used 30 trials of hyperparameter tuning with a random search. The area under the curve value of the FROC curve, with an upper limit of 10 FPs per case, was an evaluation criterion. The tuned hyperparameters were the learning rate of the SGD ($10^{-4}$ to $10^{-2}$) and batch size (2 to 64, step: 2). The number of epochs was set to 100. In the performance evaluation, a lesion candidate was classified as a TP if the label of brain metastasis had at least one voxel of the lesion candidate. This criterion is equivalent to that used in previous studies.^46^^,^^54^^,^^56^ These judgments were automatically processed.

### 10.3 Liver Lesion Classification

We used 40 trials of hyperparameter tuning with a random search, using accuracy as an evaluation criterion. The tuned hyperparameters included the learning rate of SGD ($10^{-4}$ to $10^{-2}$), batch size (2 to 64, step: 2), and dropout ratio (0 to 0.5). The number of epochs was set to 100.

## 11 Appendix D: Hyperparameters of Each Method

This section describes the hyperparameters of each method. In EC, the number of epochs was set to 100, considering that in Centralized, the number of epochs was fixed at 100. In FL, the number of epochs per round was set to 1, and the number of rounds was 100. In FL + FT and FL + FFT, the number of tuning epochs was set to 10, considering the number of tuning phase epochs in FedRep, as discussed later. The target layers of the FT were the same as those of the head in FedRep. In FedRep, the numbers of head epoch(s)/round and body epoch(s)/round were both set to 1. In addition, the number of tuning epoch(s) was fixed at 10.^17^ The division of the head and body was defined as follows. Regarding the 3D U-Net used in cerebral aneurysm detection, based on a previous study,^62^ the decoder, deepest block, and final block of the 3D U-Net were set as the head, whereas the remainder constituted the body. Regarding the SSD used for brain metastasis detection, the classification and regression layers were set to the head, with the remainder as the body. Regarding the 3D ResNet-18+CBAM used in liver lesion classification, the final fully connected layer was set as the head, and the remaining layers were set as the body. In FedBABU, the number of body epoch(s)/round was set to 1. In addition, the number of tuning epoch(s) was fixed at 10, as in FedRep. The model was likewise divided into body and head, following the same structure as that of FedRep. In APFL, the number of epoch(s)/round was set to 1, and the value of α was set to 0.25, 0.5, 0.75, and an adaptive value from 0.01, as in the original study.^21^ In Ditto and Improved Ditto, the values of $E_{\mathrm{global}}$ and $E_{\mathrm{personalized}}$ were both set to 1.

## Data Availability

Owing to ethical restrictions imposed by the Research Ethics Committee of the Faculty of Medicine of the University of Tokyo, cerebral aneurysm and liver lesion datasets are not publicly available. The original data from the OpenBTAI dataset^47^ are available in the Figshare Repository.^63^ In addition, the source code of our proposed method as applied to the brain metastasis detection task has been made publicly available at the following GitHub repository: https://github.com/utrad-ical/ImprovedDitto
